# Unveiling (−)‐Englerin A as a Modulator of L‐Type Calcium Channels

**DOI:** 10.1002/anie.201604336

**Published:** 2016-07-08

**Authors:** Tiago Rodrigues, Florian Sieglitz, Víctor J. Somovilla, Pedro M. S. D. Cal, Antony Galione, Francisco Corzana, Gonçalo J. L. Bernardes

**Affiliations:** ^1^ Instituto de Medicina MolecularFaculdade de Medicina da Universidade de LisboaAv. Prof. Egas Moniz1649-028LisboaPortugal; ^2^ Department of PharmacologyUniversity of OxfordMansfield RoadOX1 3QTOxfordUK; ^3^ Departamento de Química, Centro de Investigación en Síntesis QuímicaUniversidad de la Rioja26006LogroñoSpain; ^4^ Department of ChemistryUniversity of CambridgeLensfield RoadCB2 1EWCambridgeUK

**Keywords:** chemical biology, molecular recognition, natural products, target prediction, voltage-gated calcium channels

## Abstract

The voltage‐dependent L‐type Ca^2+^channel was identified as a macromolecular target for (−)‐englerin A. This finding was reached by using an unprecedented ligand‐based prediction platform and the natural product piperlongumine as a pharmacophore probe. (−)‐Englerin A features high substructure dissimilarity to known ligands for voltage‐dependent Ca^2+^ channels, selective binding affinity for the dihydropyridine site, and potent modulation of calcium signaling in muscle cells and vascular tissue. The observed activity was rationalized at the atomic level by molecular dynamics simulations. Experimental confirmation of this hitherto unknown macromolecular target expands the bioactivity space for this natural product and corroborates the effectiveness of chemocentric computational methods for prioritizing target‐based screens and identifying binding counterparts of complex natural products.

Natural products have traditionally served as a privileged source of chemical matter for interrogating biological systems in chemical biology and drug‐discovery programs.^1^ Their intricate architectures often allow the exploration of innovative scaffolds in drug‐relevant chemical space that is not covered by synthetic small molecules.^2^ However, factual knowledge of macromolecular targets remains elusive for most natural products, thus hindering the deployment of rational approaches for lead and chemical‐probe development. Similarly, identification of off‐targets for natural products may decisively influence their validation as chemical probes,^3^ and improve the understanding of complex polypharmacology networks in drug discovery.^4^ Herein, we disclose the voltage‐dependent Ca^2+^ L‐type (Ca_v_1.2) channel as a macromolecular target for the cancer‐selective compound (−)‐englerin A [(−)‐EA; Figure 1]. The (−)‐EA/Ca_v_1.2 binding relationship was expeditiously recognized from an unprecedented cross‐natural‐product target‐inference approach, using a small molecule with similar pharmacophore features to (−)‐EA as a tool compound, despite substructural divergence. (−)‐EA presents high affinity for the dihydropyridine binding site in Ca_v_1.2 channels, and potent calcium‐modulating effects in muscle cell‐ and tissue‐based assays. Molecular dynamics (MD) simulations further support the biology findings, and can be used to rationalize key features of Ca_v_1.2 recognition by (−)‐EA.

**Figure 1:**
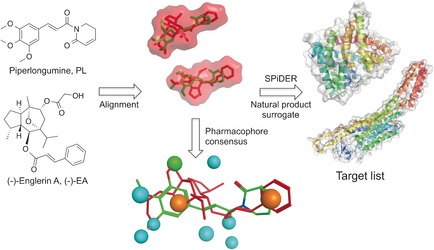
Computational target prediction to identify off‐targets for (−)‐EA. (−)‐EA and its volume are depicted in red and piperlongumine in green. A pharmacophore consensus generated with Molecular Operating environment (MOE) is shown. Orange spheres: aromatic/hydrophobic features, blue spheres: hydrogen‐bond acceptor or its projected donor, green sphere: hydrophobic feature. SPiDER performs ligand‐based target prediction according to topological descriptors. Images were generated with PyMOL (Schrödinger LLC) or MOE.

To complement proteomic methods, chemical‐structure‐driven algorithms may be swiftly deployed for steering biology research efforts and target identification.^5^ Specifically, the online SPiDER tool^6^ employs topological pharmacophore^7^ and physicochemical descriptors (Chemical Computing Group, Montreal, Canada) to qualitatively infer binding relationships between query ligands and macromolecular targets. A statistical significance estimate (*p* value) is derived for individual target predictions to support the confidence of the prediction. Importantly, the “fuzziness” of the employed descriptor renders this algorithm ideal to predicting targets for natural products.6b,6c Furthermore, target inference from virtual natural product derived fragments has been successfully achieved.6b

(−)‐EA is a sesquiterpene extracted from *Phyllanthus engleri* with selective and nanomolar‐potent cytotoxicity against renal cell carcinomas.^8^ Its remarkable effects have recently been ascribed to discriminating activation of the transient receptor potential canonical 4/5 (TRPC4/5) channels, leading to intracellular Ca^2+^ overload and cell death.^9^ Considering the current high interest in (−)‐EA as a chemical probe/lead for anticancer drug discovery,^10^ and taking into account that drug‐like molecules have been computationally predicted to engage approximately 10 drug targets,6a we initiated a pioneering program to rationally retrieve off‐targets and explore the underlying polypharmacological traits of (−)‐EA (Figure 1).

While the publicly available software tools SEA,^11^ SuperPred,^12^ and PASS^13^ did not afford confident target predictions, the online SPiDER tool6a suggested only four targets with confidence (Tables S2–4 in the Supporting Information). Indeed, the result highlights unique structural and pharmacophore features of (−)‐EA, and supports its documented renal cancer selective cytotoxicity.^11^ The result equally suggests that pharmacophore‐based descriptors may be better suited to predict targets for complex natural products compared to substructure‐based descriptors. Significantly, SPiDER retrospectively predicted TRP channels with confidence, thus corroborating the accuracy of the prediction algorithm.

Building on these encouraging results and considering that a limited array of macromolecular counterparts had been confidently predicted for (−)‐EA with state‐of‐the‐art prediction tools, we hypothesized that a fragment‐ and lead‐like natural product could serve as a pharmacophore surrogate to yield target predictions easily transferrable to (−)‐EA. Simplification of parent natural product architectures has previously been shown to afford synthetically tractable entities with modest yet sustained bioactivity.^14^ Hence, for our chemical‐genomics‐inspired target‐prediction program, we identified the fragment‐like anticancer agent piperlongumine (PL, Figure 1) as a suitable chemical tool. Piperlongumine is isolated from *Piper longum* and selectively kills cancer cells by increasing the level of reactive oxygen species.^15^ Flexible ligand alignment between (−)‐EA and PL (Figure 1) corroborated the similar shapes and potentially shared aromatic, hydrophobic, and hydrogen‐bond‐acceptor features for molecular recognition, despite their profound substructural dissimilarity (Tanimoto index=0.16, ECFP4 fingerprint). Target predictions for PL with SPiDER afforded 11 confidently predicted binding partners (Supporting Table S5), which is in line with the previously reported average for small molecules.6a The data fully support the rationale for our approach, since both natural products are confidently predicted to bind to integrins and engage TRP channels. With SPiDER target predictions in hand, we profiled both PL and (−)‐EA against the Ca_v_1.2 channels. A radioligand displacement assay revealed a modest affinity of PL for the dihydropyridine binding pocket (19 % binding inhibition at 50 μm). On the other hand, (−)‐EA showed high affinity at 10 μm (72 % radioligand displacement), while exhibiting selectivity over the remaining binding sites (Figure 2 a). (−)‐EA showed concentration‐dependent radioligand displacement and potent cooperative binding to the dihydropyridine binding site (Hill slope>2, *K*_i_=5.7±0.4 μm, Figure 2 b and the Supporting Information).

**Figure 2:**
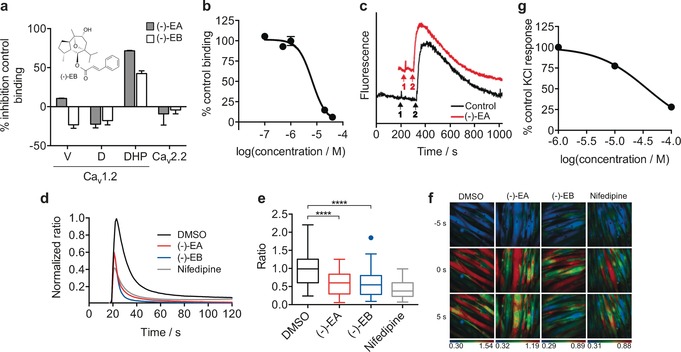
Engagement of Ca_v_1.2 by (−)‐Englerin A [(−)‐EA]. a) Screening of (−)‐EA and (−)‐Englerin B [(−)‐EB] in a radioligand displacement assay against three different binding sites (verapamil, diltiazem, and dihydropyridine) of Ca_v_1.2 and Ca_v_2.2 at 10 μm. Error bars reflect the range of two replicates. V*=*Verapamil; D=Diltiazem; DHP=Dihydropyridine. b) IC_50_ curve of (−)‐EA against the dihydropyridine binding site (*K*_i_=5.7±0.4 μm; Control: Nitrendipine, *K*_i_=0.19 nm (*n*Hill=2.1)). Error bars reflect the range of two replicates. c) Exemplary trace of the (−)‐EA screen against two‐pore channels 1/2 (TPC1/2). Event 1 corresponds to the addition of either DMSO (0.1 %) or (−)‐EA (10 μm) and event 2 corresponds to addition of the Ca^2+^‐releasing agent NAADP (100 nm). The data show no significant difference to the DMSO control. d) (−)‐EA and (−)‐EB (6 μm) inhibit KCl‐induced Ca^2+^ influx in rat cardiomyocytes. The normalized average of Fura‐2 AM ratio of KCl‐responding polynucleated H9C2 cells was monitored for DMSO (*n=*61), (−)‐EA (*n=*58), (−)‐EB (*n=*66), and nifedipine (*n=*58). Non‐responding cells (<0.1 ratio increase) were not included in the analysis. e) A box plot of peak maxima after KCl stimulation of H9C2 cells (control: 1 % DMSO; (−)‐EA, and (−)‐EB: 6 μm in 1 % DMSO; Nifedipine: 10 μm in 1 % DMSO). One‐way ANOVA amongst all response maxima after KCl stimulation: **** *p*<0.0001. f) Live muscle cell calcium imaging. Exemplary images 5 s before KCl‐induced [Ca^2+^]_i_ maximum, at maximum and 5 s after KCl‐induced [Ca^2+^]_i_ maximum (post‐treatment). The Fura‐2 AM ratio is color‐coded as indicated (blue: low ratio, red: high ratio). g) Antagonism by (−)‐EA in endothelium‐denuded isolated rat thoracic aorta (EC_50_=37±1 μm). Error bars reflect the range of two replicates.

From a pharmacophore perspective, the difference in activity between PL and the complex (−)‐EA can be retrospectively explained on the basis of features unmet by the fragment‐like PL that may be critical for Ca_v_1.2 binding, for example, a H‐bond donor (Figure 1). (−)‐EA significantly expands known Ca_v_1.2 channel ligand space, since its nearest neighbour in ChEMBL v20, CHEMBL201 599, presents low substructure similarity (Tanimoto index=0.22, ECFP4 fingerprint; Figure S3 in the Supporting Information).

The results highlight the accuracy of the prediction algorithm and the cross‐natural‐product target‐inference strategy to identify unexpected ligand–target relationships in the absence of substructural similarity to known target modulators. The range of affinities of (−)‐EA towards different binding sites and the lack of detection of adducts upon co‐incubation with N‐ and C‐protected cysteine suggest specific non‐covalent binding to Ca_v_1.2 and an absence of false‐positive readouts. Dynamic light scattering experiments at relevant concentrations support sample polydispersity with an absence of artifactual binding (Figures S1, S2). (−)‐EA does not present measurable binding affinity towards the N‐type Ca^2+^ channel, nor does it engage two‐pore calcium channels 1/2 (TCP1/2), which demonstrates its selectivity within a panel of selected voltage‐gated Ca^2+^ channels (Figure 2 a, c and Figure S4).

To assess the biological relevance of the binding of (−)‐EA to Ca_v_1.2, we performed calcium imaging using the rat cardiomyocytic cell line H9C2. H9C2 cells lack expression of the (−)‐EA targets TRPC4/5, while expressing Ca_v_1.2 upon differentiation. Indeed, incubation with (−)‐EA in concentrations as high as 10 μm did not induce any calcium influx in undifferentiated H9C2 cells, thus suggesting that the cells are devoid of TRPC4/5 expression (data not shown). We induced H9C2 differentiation in low serum and 10 nm retionic acid as previously described.^16^ After five days of incubation, most H9C2 cells appeared polynucleated and expressed Ca_v_1.2, as assessed by western blots probed with anti‐Ca_v_1.2 antibodies (Figure S5). Polynucleated H9C2 cells showed a strong Ca^2+^ influx when depolarized with 60 mm KCl, as measured by the calcium‐sensitive probe Fura‐2 AM. A 45 min pre‐treatment with 6 μm (−)‐EA or 10 μm nifedipine (control) significantly reduced the KCl‐induced Ca^2+^ influx (one‐way ANOVA amongst peak maxima for control [1 % DMSO, (−)‐EA (6 μm in 1 % DMSO), and nifedipine (10 μm in 1 % DMSO), *p*<0.0001; Figure 2 d–f]. These observations are in line with the hypothesis that (−)‐EA blocks Ca_v_1.2 channels. As an orthogonal functional assay, we screened (−)‐EA in endothelium‐denuded isolated rat thoracic aorta. To avoid confounding readouts, we blocked all G‐protein‐coupled receptors that can interfere with Ca^2+^ signalling, that is, the α‐ and β‐adrenergic, histamine H_1_, muscarinic, and serotonin 5‐HT_2_ receptors. As expected, KCl induced a concentration‐dependent tissue contraction, which was inhibited by nitrendipine. Similarly, (−)‐EA blocked the KCl‐induced mechanical effect in a concentration‐dependent manner (EC_50_=37±1 μm; Figure 2 g), thus corroborating the previously observed antagonism.

To provide a molecular rationale for this experimental data, we performed MD simulations on the (−)‐EA/Ca_v_1.2 channel complex. Considering the fact that there are no reported crystal structures for these channels, we used the open‐state Ca_v_1.2 homology model reported by Zhorov et al.^17^ and performed molecular docking of (−)‐EA into the dihydropyridine binding site with AutoDock Vina.^18^ The binding pose with the best score was subjected to MD simulations with AMBER12 in explicit water and Ca^2+^ ions over 125 ns in a DOPC bilayer. The MD simulation trajectory suggests that the ligand/Ca_v_1.2 complex is stabilized by hydrophobic interactions and a hydrogen bond. A CH−π interaction is predicted between the phenyl ring in (−)‐EA and the methylene of the Phe202 side chain. Furthermore, the hydroxy group of the ligand establishes a hydrogen bond, populated approximately 92 % of the total trajectory time, with the backbone of Leu121 (Figure 3 a). Interestingly, the hydrogen bond is predicted to be transiently broken without major shifts in the pose of (−)‐EA, thus rationalizing the importance of hydrophobic contacts for stabilization of the ligand/receptor complex (Figure 3 b). No significant interactions with the binding pocket were observed in MD simulations starting from diverse lower‐scored (−)‐EA poses or for piperlongumine (Figure S6).

**Figure 3:**
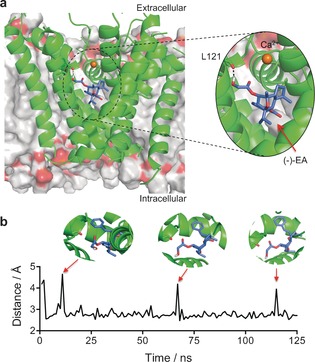
Molecular modelling of the (−)‐EA/Ca_v_1.2 complex. a) Stabilizing interactions found through the 125 ns MD simulation between (−)‐EA and the Ca_v_1.2 homology model. Hydrogen bonding with leucine 121 (backbone) is populated 92 % of the total trajectory time. (−)‐EA is depicted in purple, the Ca_v_1.2 channel in green, and the DOPC bilayer in gray. b) Monitoring of the L121‐(−)‐EA interaction distance over the simulation time. Images were generated with PyMOL (Schrödinger LLC).

For ascertaining the importance of the predicted hydrogen bonding between (−)‐EA and Leu121, we synthesized the glycolic acid free analogue of (−)‐EA, (−)‐Englerin B [(−)‐EB], as previously described.^19^ MD simulations with the (−)‐EB/Ca_v_1.2 channel complex predicted no hydrogen bonds over the total trajectory time. In fact, our data suggest that a hydrogen bond with Leu121 might be important but not the sole determinant for the affinity of (−)‐EA, since its analogue (−)‐EB presents *K*_i_>10 μm against the dihydropyridine binding site of Ca_v_1.2, although with measurable functional effects (Figures 2 a,e). No appreciable affinity to the verapamil and diltiazem binding sites in Ca_v_1.2 and Ca_v_2.2 was found for (−)‐EB in radioligand displacement assays (Figure 2 a). Altogether, the in vitro data for both natural products are commensurate with directed and specific target interaction and corroborate the in silico rationale for molecular recognition. Thus, selected structural features of (−)‐EA may be grafted onto small molecules in ligand‐design programmes.

Natural products remain an important source of chemical matter in drug discovery. Nonetheless, their value as chemical probes requires extensive validation and exploitation of on‐/ off‐target engagement effects and systems biology. (−)‐EA has recently attracted considerable attention in cancer research. We experimentally confirmed the validity of our pioneering target‐prediction concept. We disclose functional effects of (−)‐EA on Ca^2+^ signalling pathways through Ca_v_1.2 channel modulation, and identify the relevant molecular recognition mechanisms. From a computational point of view, the successful off‐target identification can be ascribed to the topological pharmacophore descriptors and consensus self‐organizing map method implemented in SPiDER, in contrast to the substructure‐based fingerprints implemented in other tools. Whereas natural products display different architectures compared to lead‐like molecules, their potential pharmacophore features are sufficiently similar to those of drug‐ and lead‐like molecules to allow confident target inferences.

It is noteworthy that false‐positive predictions are still expected with this or related platforms, and only previously liganded macromolecules can be discovered as on‐ and off‐targets for the query natural products. That said, the results suggest an expeditious chemogenomics‐inspired platform to rationally steer screening efforts and unveil hitherto unknown pharmacology for complex chemotypes lying outside of drug‐like space and thus outside of the domain of applicability of current state‐of‐the‐art and publicly available target‐prediction tools.

Ultimately, we foresee a broad scope and prime utility of related technologies to identify unexpected, although much sought after, polypharmacology networks for intricate natural products, and to leverage the design of natural product inspired entities for chemical biology and molecular medicine.

## Supporting information

As a service to our authors and readers, this journal provides supporting information supplied by the authors. Such materials are peer reviewed and may be re‐organized for online delivery, but are not copy‐edited or typeset. Technical support issues arising from supporting information (other than missing files) should be addressed to the authors.

Supplementary
