# Supplementary material for: Unveiling (−)‐Englerin A as a Modulator of L‐Type Calcium Channels
Source: Angew Chem Int Ed Engl. 2016 Jul 8;55(37):11077–81. doi: 10.1002/anie.201604336 (PMC5042069; doi:10.1002/anie.201604336)
Supplement: Supplementary file 1 — Supplementary [file ANIE-55-11077-s001.pdf]

Angewandte Chemie
Eine Zeitschrift der Gesellschaft Deutscher Chemiker

Supporting Information

Unveiling (−)-Englerin A as a Modulator of L-Type Calcium Channels

Tiago Rodrigues[+], Florian Sieglitz[+], Víctor J. Somovilla, Pedro M. S. D. Cal, Antony Galione, Francisco Corzana,* and Gonçalo J. L. Bernardes**

anie_201604336_sm_miscellaneous_information.pdf

Table of contents

1. Experimental section **… S2**

1.1 Computational chemistry … S2

1.1.1 Target prediction … S2

1.1.2 Flexible ligand alignment … S2

1.1.3 Molecular docking … S2

1.1.4 MD simulations … S2

1.1.5 Nearest neighbour and Principal Component Analysis … S3

1.2 Biochemistry … S3

1.2.1 Radioligand displacement assays … S3

1.3 Biophysics and analytical chemistry … S4

1.3.1 Adduct formation … S4

1.3.2 Dynamic light scattering … S4

1.4 Biology … S4

1.4.1 Cell culture (H9C2) … S4

1.4.2 Intracellular calcium (Ca^{2+}_i) measurement in H9C2 rat cardiomyocytes … S4

1.4.3 Two-pore channel engagement … S5

1.4.4 Contractility assay in tissue … S5

1.5 Chemistry … S6

1.5.1 General considerations … S6

1.5.2 Synthesis … S7

2. Supplementary data **… S8**

2.1 Target predictions … S8

2.2 Dynamic light scattering … S9

2.3 Adduct formation … S10

2.4 Principal component analysis … S10

2.5 Two-pore channel assay data … S11

2.6 Cardiomyocyte assay … S12

2.7 Molecular dynamics simulations … S12

2.8 ^{1}H NMR … S13

3. References **… S14**

1. Experimental section

1.1 Computational chemistry

1.1.1 Target prediction

Target prediction with SPiDER was carried out on the publically available web server (www.modlab-cadd.ethz.ch/software/spider) as previously reported.[1] In summary, the query molecules are projected onto two self-organizing maps together with reference compounds from the COBRA database.[2] Chemical structures are processed with the "wash" function of the Molecular Operating Environment (MOE, Chemical Computing Group, Montreal, Canada), prior to description with the CATS2[3] pharmacophore and MOE descriptors. Predictions are carried out by calculating the Euclidean distance of the query molecule to the reference compounds in COBRA. The output comprises target families at a confidence level of $p < 5\%$. The aforementioned distances are converted to p values, according to a pre-calculated background distribution of distances between molecules annotated to bind different targets. The arithmetic average of these p values serves as confidence score for the target prediction. With the background distribution of confidence scores, each prediction can be associated with another p value that indicates the statistical significance of the prediction.[1, 4]

1.1.2 Flexible ligand alignment

(-)-Englerin A, (-)-EA, and piperlongumine were "washed" with default MOE v2014.9 (Chemical Computing Group, Montreal, Canada) options. The Amber12EHT (Extended Hückel Theory) force-field was used to parameterize both natural products prior to flexible alignment, (Iteration limit = 20; energy cutoff = 15 kcal/mol; stochastic conformational search allowed).

1.1.3 Molecular docking

Ligands were docked into L-type calcium channels model reported by Tikhonov *et al*[5] with the aid of of AutoDock Vina 1.1.2.[6] The predicted binding energies ranged from -6.5 to -8.0 kcal·mol^{-1}. The Autogrid grid point spacing was set at 0.375 Å, center coordinates of the grid box were -6.9, 1.0, -7.4 (x, y, z), and number of grid points in *xyz* was 116, 92, 82, respectively. All allowed torsional bonds were considered rotatable. The 3D-structures of the docked ligand to the channel system with the best binding energies were optimized performing MD simulations.

1.1.4 MD simulations

Bilayers of 1,2-dioleoyl-sn-glycero-3-phosphocholine lipid, together with L-type calcium channel, ligand, TIP3P water molecules and KCl to neutralize the system, were constructed using the CHARMM Membrane Builder GUI[7] and converted to Lipid14 PDB format with the *charmmlipid2amber.x* script included in Amber14.[8] The full system was minimized for 10000 steps, of which the first 5000 steps used the steepest descent method and the remaining steps used the

conjugate gradient method.[9] The system was then heated from 0 K to 100 K using Langevin dynamics[10] for 5 ps at constant volume, with weak restraints on the lipid (force constant 10 kcal mol^{-1} $Å^{-2}$). Following this, the volume was allowed to change freely and the temperature increased to 300K with a Langevin collision frequency of γ=1.0 ps^{-1}, and anisotropic Berendsen regulation[11] (1 atm) with a time constant of 2 ps for 100 ps. The same weak restraint of 10 kcal mol^{-1} $Å^{-2}$ was maintained on the lipid molecules. Constant pressure and constant temperature (NPT) runs were performed on the different systems using the AMBER 14. Bonds involving hydrogen were constrained using the SHAKE algorithm, allowing a 2 fs time step. Structural data was recorded every 10 ps. PME was used to treat all electrostatic interactions with a real space cutoff of 10 Å. A long-range analytical dispersion correction was applied to the energy and pressure. All simulations were performed at constant pressure of 1 atm and constant target temperature. Temperature was controlled by the Langevin thermostat, with a collision frequency of γ=1.0 ps^{-1}. Pressure was regulated by the anisotropic Berendsen method (1 atm) with a pressure relaxation time of 1.0 ps. Each system was simulated for 125 ns.

1.1.5 Nearest neighbour and Principal Component Analysis

L-type channel modulators (IC_{50} < 50 μM) were collected from ChEMBL v20. Extended connectivity fingerprints, radius 4 (ECFP4), and Tanimoto indices were calculated with RDKit native nodes implemented in KNIME (www.knime.org). RDKit native nodes were employed to calculate topological descriptors, including molecular weight, logP, number of hydrogen bond donors / acceptors, fraction of sp^3 bonds, number of rings, and atom count. Data was submitted to Principal Component Analysis (PCA) and PC1 *vs.* PC2 plotted in GraphPad Prism.

1.2 Biochemistry

1.2.1 Radioligand displacement assays

Radioligand displacement assays were performed at Cerep, SA (Celle l'Evescault, France) on a fee-for-service basis. The voltage-gated calcium (Ca^{2+}) channel (L-type) dihydropyridine site (Ref 0161),[12] diltiazem site (Ref 0162),[13] verapamil site (Ref 0163),[14] and N-type (Ref 0164)[15] were assayed as described in Table S1.

Table S1. Ca^{2+} L-type (dihydropyridine, diltiazem and verapamil binding sites) and N-type channel assay protocols.

Ca^{2+} channel	Source	Ligand	Incubation	Detection method
Dihydropyridine site	Rat cerebral cortex	[^{3}H]nitrendipine (0.1 nM)	90 min, r.t.	Scintillation counting
Diltiazem site	Rat cerebral cortex	[^{3}H]diltiazem (15 nM)	120 min, r.t.	Scintillation counting
Verapamil site	Rat cerebral cortex	[^{3}H]D888 (3 nM)	120 min, r.t.	Scintillation counting
N-type	Rat cerebral cortex	[^{125}I] ω-conotoxin GVIA (0.001 nM)	30 min, r.t.	Scintillation counting

Controls: Dihydropyridine site: Nitrendipine, K_i = 0.19 nM (nHill = 2.1); Diltiazem site: Diltiazem, K_i = 12 nM (nHill = 1.3); Verapamil site: D600, K_i = 11 nM (nHill = 0.6); N-type: ω-conotoxin GVIA, K_i = 0.003 nM (nHill = 1.2).

1.3 Biophysics and analytical chemistry

1.3.1 Adduct formation

N- and C-protected cysteine (Boc-Cys-OMe, 100 µM) was incubated with (-)-EA (100 µM) in TRIS-HCl buffer (50 mM, pH 7.4) at 22 ºC for 60 minutes, simulating the screening assay conditions.[13] Adduct formation was monitored by direct injection of a sample into a mass spectrometer (LCQ Fleet, Thermo Scientific).

1.3.2 Dynamic light scattering

Particle size distributions were measured at a wavelength of 633 nm and a scattering angle of 173° with linear spacing of the correlation time in a Malvern ZetaSizer ZSP. Data was collected at 25 ºC and at a voltage of 150 V prior to analysis by a digital autocorrelation software (Zetasizer Software). The effective diameter was intensity-based calculated. Average values are reported for the zeta potentials (n = 3).

1.4 Biology

1.4.1 Cell culture (H9C2)

H9C2 cells (ATCC) were grown to 90% confluency in DMEM (Gibco) supplemented with 10% FBS (Gibco), GlutaMax (Gibco), MEM and NEAA (Gibco), 4.5 g/l glucose and 0.11 g/l sodium pyruvate. Differentiation was initiated in the same medium except for 1% FBS and 10nM retinoic acid for 5 days as previously described.[16]

1.4.2 Intracellular calcium (Ca^{2+}_i) measurement in H9C2 rat cardiomyocytes

Intracellular calcium measurements were performed with Fura-2 AM (Life Technologies) and modified from previously described studies.[17] In short, 1 hour before the measurement, the H9C2 medium was changed to calcium recording buffer (CRB) (125 mM NaCl, 2 mM $MgCl_2$, 5 mM KCl, 2 mM $CaCl_2$, 10 mM glucose, 2 % FBS, 20 mM HEPES, pH 7.4). Cells were loaded with 5 µM Fura-2

AM for 45 minutes. After 15 minutes the respective treatments were added to the cells (final of 1 % DMSO (control), 6 µM (-)-EA and 10 µM nifedipine). After Fura-2 AM incubation, cells were washed thrice with PBS and allowed to re-adjustment for 15 minutes in CRB (plus the respective treatment). Fura-2 AM emissions from 340 nm and 380 nm excitation were recorded for 1 minute before cells were depolarized by addition of the same CBS volume of KCl-buffer (15 mM NaCl, 2 mM $MgCl_2$, 115 mM KCl, 2 mM $CaCl_2$, 10 mM Glucose, 2 % FBS, 20 mM HEPES pH 7.4 + the respective treatment) to the imaging chambers (final KCl concentration 60 mM). Image analysis was done using MetaFluor Analyst software. The statistical analysis was performed with GraphPad Prism.

1.4.3 Two-pore channel engagement

Sea urchin eggs from *Lytechinus pictus* were harvested by intracoelomic injection of 0.5 M KCl, collected in ASW (artificial seawater: 435 mM NaCl; 40 mM $MgCl_2$; 15 mM $MgSO_4$; 11 mM $CaCl_2$; 10 mM KCl; 2.5 mM $NaHCO_3$ and 20 mM Tris base pH 8.0) and de-jellied by passage through 100 µm nylon mesh (Millipore). Egg homogenates were prepared at 4 °C and stored at -80 °C. Essentially, eggs were washed four times in Ca^{2+}-free ASW (the first two washes containing 1 mM EGTA) and then washed in GluIM (gluconate intracellular-like medium: 250 mM potassium gluconate; 250 mM *N*-methylglucamine; 20 mM Hepes and 1 mM $MgCl_2$, pH 7.2). Eggs were then homogenized in GluIM supplemented with 2 mM Mg-ATP, 20 units/mL creatine phosphokinase, 20 mM phosphocreatine and a complete TM EDTA-free protease inhibitor tablet (Roche). The homogenate (50%, v/v) was centrifuged at 13000 *g* at 4 °C for 10 s and the supernatant stored at -70 °C. On the day of use, an aliquot (500 µL) of homogenate was sequentially diluted in equal volumes of GluIM (containing the ATP regenerating system above) over a period of 3 h at 17 °C to give a 2.5% (v/v) final concentration.

Fluorimetry of 600 µL aliquots of sea urchin egg homogenate was conducted at 17 °C in a microcuvette containing a magnetic stir bar mounted in a PerkinElmer LS-50B fluorimeter. Ca^{2+} release was routinely measured in homogenates with 3 µM Fluo-3 (excitation 506 nm, emission 526 nm) and expressed as arbitrary fluorescence units. Addition of (-)-EA or vehicle was made by adding 0.6 µL of stock solution to give the final test concentration. The traces are representative of at least three experiments.

1.4.4 Contractility assay in tissue

The assay was performed at Cerep, SA (Celle l'Evescault, France) on a fee-for-service basis according to the procedure of Okamiya *et al.*[18] Rings of rat thoracic aorta denuded of endothelium were suspended in 20 mL organ baths filled with an oxygenated (95% O_2 and 5% CO_2) and pre-warmed (37 °C) physiological salt solution of the following composition: NaCl 118.0 mM, KCl 4.7 mM, $MgSO_4$ 1.2 mM, $CaCl_2$ 2.5 mM, KH_2PO4 1.2 mM, $NaHCO_3$ 25 mM and glucose 11.0 mM (pH 7.4). High K^+ solution (60 mM KCl) was prepared by equimolar replacement of NaCl with KCl.

Phenoxybenzamine (0.1 μM), propranolol (1 μM), pyrilamine (1 μM), atropine (1 μM) and methysergide (1 μM) were also present throughout the experiments to block the α-adrenergic, β-adrenergic, histamine H_1, muscarinic and 5-HT_2 receptors, respectively. The tissues were connected to force transducers for isometric tension recordings. They were stretched to a resting tension of 2 g, and then allowed to equilibrate for 60 minutes during which time they were washed repeatedly and the tension readjusted. The experiments were carried out using semi-automated isolated organ systems possessing eight organ baths, with multichannel data acquisition. The parameter measured is the maximum change in tension induced by each compound concentration. Two independent measurements were conducted.

Evaluation of agonist activity

The tissues were exposed to a high K^+ solution (60 mM KCl) to verify responsiveness and to obtain a control contractile response. Following washings and recovery of the basal tension, the tissues were exposed to increasing concentrations of the test compound or KCl. The concentrations were added cumulatively and each was left in contact with the tissues until a stable response was obtained or for a maximum of 30 minutes. If an agonist-like response (contraction) was obtained, the reference antagonist nitrendipine (0.03 μM) was tested against the highest concentration of the compound to confirm the involvement of the L-type Ca^{2+} channels in this response.

Evaluation of antagonist activity

The tissues were exposed to a high K^+ solution (60 mM KCl) to obtain a control contractile response. After stabilization of the KCl-induced response, the tissues were exposed to increasing concentrations of the test compound or the reference antagonist nitrendipine. The concentrations were added cumulatively and each was left in contact with the tissues until a stable response was obtained or for a maximum of 30 minutes. An inhibition of the KCl-induced response by the test compound indicates an antagonist activity at the L-type Ca^{2+} channels.

1.5 Chemistry

1.5.1 General considerations

(-)-Englerin A was purchased from PhytoLab, Germany and solvents and reagents were purchased from Sigma Aldrich, Fluka or Merck and used without further purification. Preparative TLC plates were purchased from Merck. The proton nuclear magnetic resonance (^1H NMR) spectrum was recorded on a Bruker Avance 300 (300 MHz). All chemical shifts are quoted on the δ scale in ppm using residual solvent peaks as the internal standard. Coupling constants (*J*) are reported in Hz with the following splitting abbreviations: d = doublet, dd = doublet of doublets, m = multiplet. Mass spectrometry analyses were operated in positive-ion mode with ESI. Nominal and exact m/*z* values are reported in Dalton.

1.5.2 Synthesis

(-)-Englerin B was synthesized as previously described.[19] (-)-Englerin A (1 molar eq.) and K_2CO_3 (7 molar eq.) were dissolved in MeOH (1 L / mmol). The solution was stirred for 1 h at room temperature, before adding a saturated aqueous solution of NH_4Cl (2.5 L / mmol) and extracting

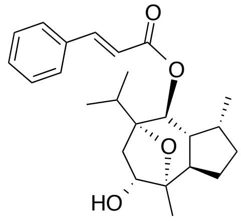

with dichloromethane (3×50 mL). The combined extracts were dried over $MgSO_4$ and the solvent evaporated under vacuum. The crude mixture was purified via preparative TLC (AcOEt : hexanes 1:4) to afford pure (-)-Englerin B as a colourless oil, 94%. ^{1}H NMR (300 MHz, CD_3OD): δ 0.86 (3H, d, *J* = 6.9 Hz), 0.90 (3H, d, *J* = 7.2 Hz), 0.97 (3H, d, *J* = 6.9 Hz), 1.00-1.30 (7H, m), 1.50-2.10 (7H, m), 2.58 (1H, dd, *J* = 12.0 and 7.8 Hz), 3.99 (1H, m), 4.01 (1H, d, *J* = 7.8 and 3.0 Hz), 5.02 (1H, d, *J* = 10.2 Hz), 6.45 (1H, d, *J* = 16.0 Hz), 7.35 (3H, m), 7.55 (2H, m), 7.62 (1H, d, *J* = 16.0 Hz).

2. Supplementary data

2.1 Target predictions

No targets were predicted for (-)-EA using SuperPred.

Table S2. SPiDER target predictions for (-)-EA.

Target family	*p* value
Microtubules[a]	0.033
Integrins	0.048
Glucocorticoid receptor	0.048
Transient Receptor Potential (TRP) channel	0.050

[a] No effect on tubulin polymerization was observed for (-)-EA (50 μM) (data not shown).

Table S3. PASS target predictions for (-)-EA.

Target	Pa	Pi
Antineoplastic	0.948	0.004
Prostate cancer treatment	0.613	0.005
Antieczematic	0.637	0.070
Phosphatase inhibitor	0.604	0.045
Antimetastatic	0.511	0.018
Immunosuppressant	0.501	0.042
CYP3A2 substrate	0.521	0.064
Apoptosis agonist	0.492	0.042
Antipruritic, allergic	0.487	0.046
Myc inhibitor	0.457	0.022

Table S4. SEA target predictions for (-)-EA.

Target	E value
DNA polymerase beta	7.13×10^{-12}
P-glycoprotein 1	6.58×10^{-5}
Protein kinase C delta	5.84×10^{-2}
Protein kinase C delta	3.77×10^{-1}
Protein kinase C delta	3.77×10^{-1}
Protein kinase C delta	3.77×10^{-1}
P-glycoprotein 1	2.13×10^{0}

Table S5. SPiDER target predictions for piperlongumine.

Target family	*p* value
Endopeptidase (cysteine endopeptidase)	0.005
Calcium channel	0.007
Interleukin receptor	0.007
Adrenergic receptor	0.007
Integrins	0.010
Phosphodiesterase	0.013
Corticotropin-releasing factor receptor	0.024
Transient Receptor Potential (TRP) channel	0.030
Smoothened receptor	0.030
Tyrosine kinase	0.032
Adenosine receptor	0.033

2.2 Dynamic light scattering

Table S6. Results of dynamic light scattering for (-)-EA.

Concentration (µM)	Effective diameter (nm)	Y- Intercept	Zeta Potential	Mean Count Rate (kpcs)
50	465	0.92	-46.3	234
25	655	0.81	-36.6	332
12.5	383	0.83	-31.8	171
6.25	211	0.75	-8.5	288
3.12	794	0.06	-3.8	111
1.56	695	0.10	-6.2	187
0	0	0.14	----	----

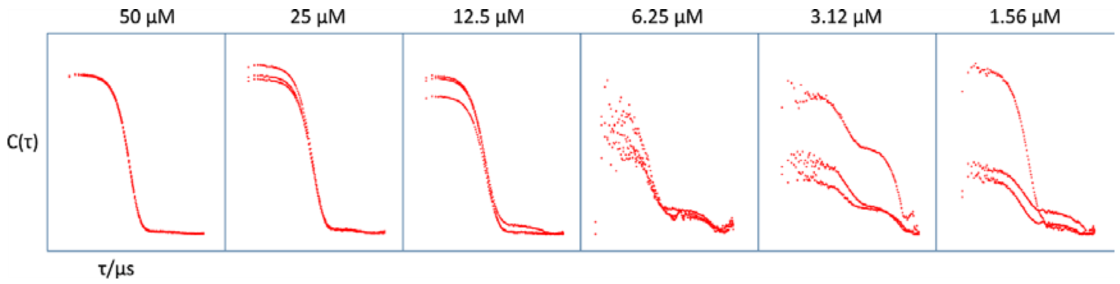

Figure S1. Correlation functions C(τ) from dynamic light scattering. Data were obtained for six different concentrations of (-)-EA in water + 0.1% DMSO. Meaningful colloid formation is observable for (-)-EA concentrations exceeding approximately 10 µM.

2.3 Adduct formation

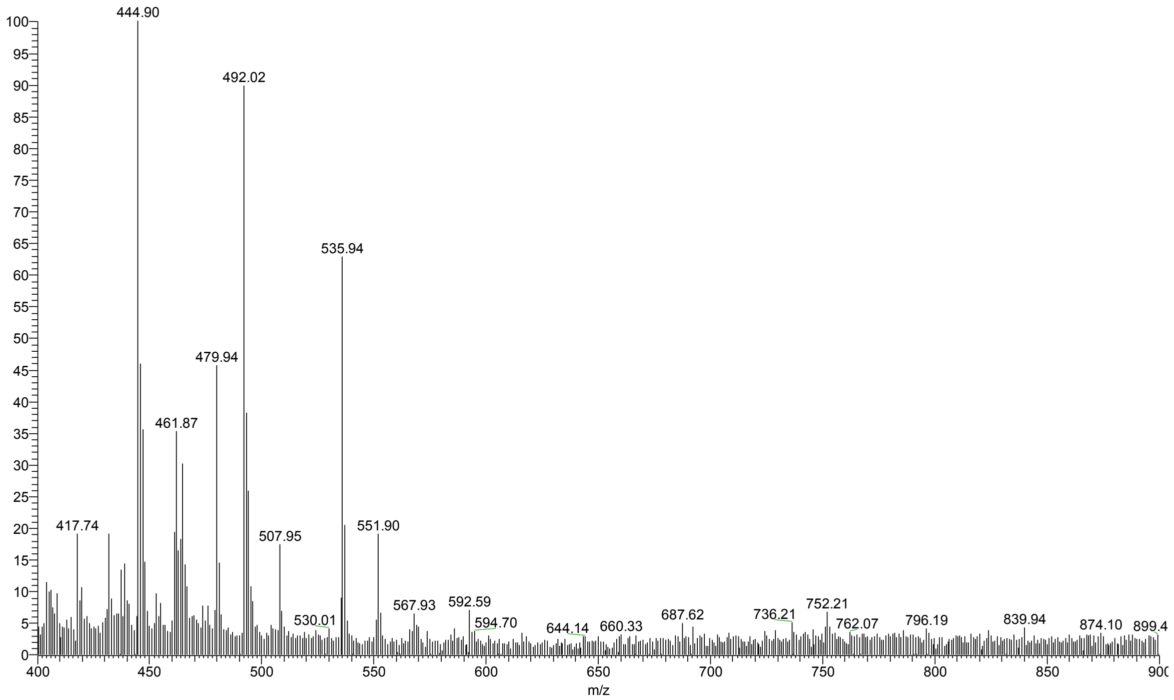

Figure S2. MS spectrum (ESI+) of (-)-EA (100 μM) and *N*- and C-protected cysteine (Boc-Cys-OMe (100 μM) dissolved in Tris.HCl (50 mM, pH 7.4), shows no adducts.

2.4 Principal component analysis

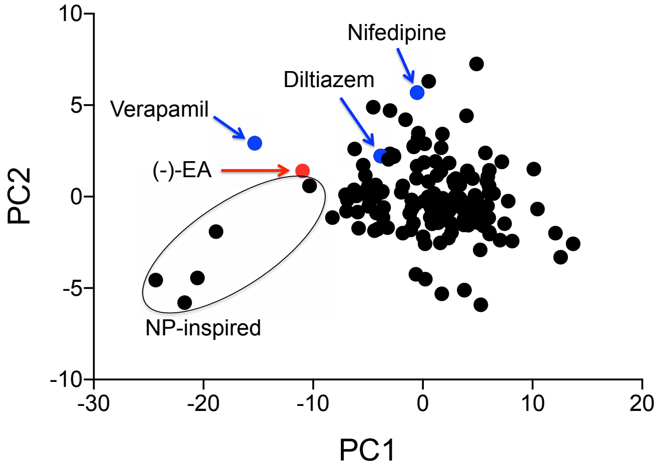

Figure S3. Principal Component Analysis of L-type Ca^{2+} channel blockers and (-)-EA. Data shows clustering of (-)-EA among known L-type Ca^{2+} channel blockers. Prototypical blockers (verapamil, diltiazem and nifedipine) and (-)-EA are depicted as blue and red dots, respectively. Natural product-inspired chemical matter[20] shares divergent topological properties to most of bioactive chemical entities.

2.5 Two-pore channel assay data

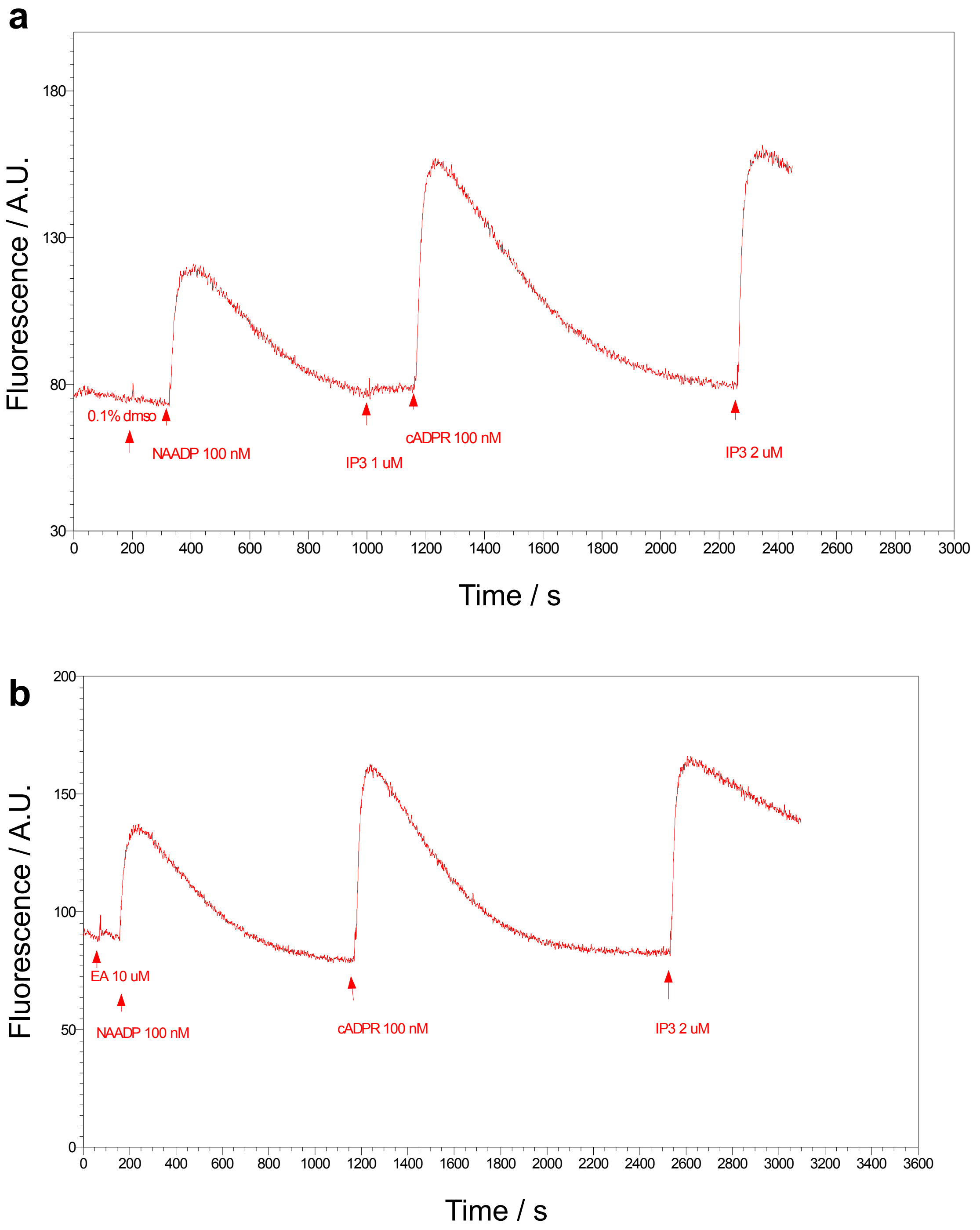

Figure S4. Exemplary uncut traces for the two-pore channel 1/2 (TPC1/2) engagement assay. (**a**) 0.1% DMSO control with time-specific addition of Ca^{2+}-releasing agents NAADP (100 nM), IP3 (1 and 2 µM) and cADPR (100 nM). (**a**) Assay of (-)-EA (10 µM) with time-specific addition of Ca^{2+}-releasing agents NAADP (100 nM), cADPR (100 nM) and IP3 (2 µM). (-)-EA shows no effect on NAADP-, IP3- and cADPR-mediated Ca^{2+} release.

2.6 Cardiomyocyte assay

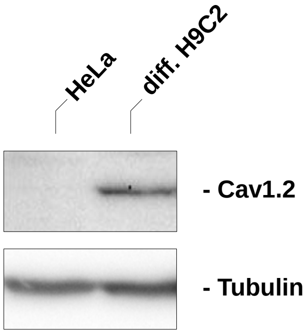

Figure S5. Western Blot probed with anti-Ca_v1.2a antibodies (1:200, Alomone Labs) and anti-tubulin antibodies (1:500, Sigma). Negative control: HeLa cell lysate.

2.7 Molecular dynamics simulations

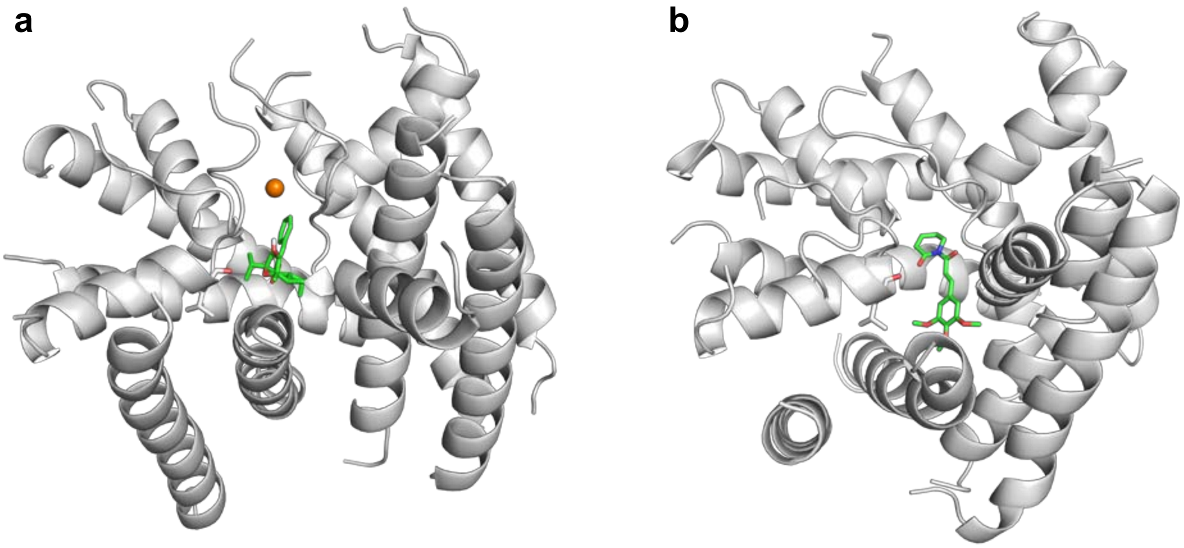

Figure S6. Representative frames of the MD data for (-)-englerin B (a) and piperlongumine (b). The DOPC bilayer and water molecules were omitted for clarity. Images were generated with PyMOL (Schrödinger LLC).

2.7 ^{1}H NMR

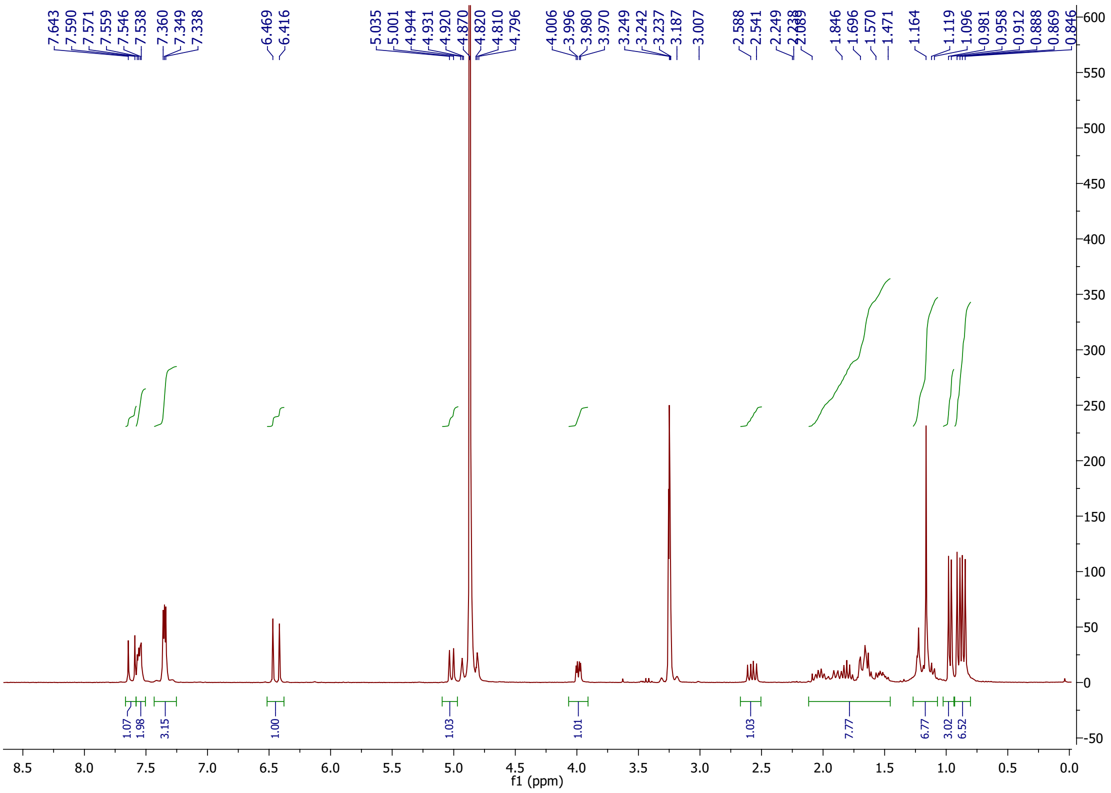

Figure S7. ^{1}H NMR spectrum for (-)-englerin B in CD_3OD.

3. References

[1] D. Reker, T. Rodrigues, P. Schneider, G. Schneider, *Proc. Natl. Acad. Sci. U.S.A.* **2014**, *111*, 4067-4072.

[2] P. Schneider, G. Schneider, *QSAR Comb. Sci.* **2003**, *22*, 713-718.

[3] M. Reutlinger, C. P. Koch, D. Reker, N. Todoroff, P. Schneider, T. Rodrigues, G. Schneider, *Mol. Inf.* **2013**, *32*, 133-138.

[4] a) D. Reker, A. M. Perna, T. Rodrigues, P. Schneider, M. Reutlinger, B. Monch, A. Koeberle, C. Lamers, M. Gabler, H. Steinmetz, R. Muller, M. Schubert-Zsilavecz, O. Werz, G. Schneider, *Nat. Chem.* **2014**, *6*, 1072-1078; b) T. Rodrigues, D. Reker, J. Kunze, P. Schneider, G. Schneider, *Angew. Chem. Int. Ed.* **2015**, *54*, 10516-10520.

[5] D. B. Tikhonov, B. S. Zhorov, *J. Biol. Chem.* **2009**, *284*, 19006-19017.

[6] O. Trott, A. J. Olson, *J. Comput. Chem.* **2010**, *31*, 455-461.

[7] S. Jo, J. B. Lim, J. B. Klauda, W. Im, *Biophys. J.* **2009**, *97*, 50-58.

[8] D. A. Case, J. T. Berryman, R. M. Betz, D. S. Cerutti, I. T.E. Cheatham, T. A. Darden, R. E. Duke, T. J. Giese, H. Gohlke, A. W. Goetz, N. Homeyer, S. Izadi, P. Janowski, J. Kaus, A. Kovalenko, T. S. Lee, S. LeGrand, P. Li, T. Luchko, R. Luo, B. Madej, K. M. Merz, G. Monard, P. Needham, H. Nguyen, H. T. Nguyen, I. Omelyan, A. Onufriev, D. R. Roe, A. Roitberg, R. Salomon-Ferrer, C. L. Simmerling, W. Smith, J. Swails, R. C. Walker, J. Wang, R. M. Wolf, X. Wu, D. M. York, P. A. Kollman, AMBER14, University of San Francisco, San Francisco, USA, **2014**.

[9] W. H. Press, S. A. Teukolsky, W. T. Vetterling, B. P. Flannery, *Numerical recipes: The art of scientific computing*, 3rd ed ed., Cambridge University Press, New York, **2007**.

[10] R. Pastor, B. Brooks, A. Szabo, *Mol. Phys.* **1988**, *65*, 1409-1419.

[11] H. J. C. Berendsen, J. P. M. Postma, W. F. v. Gunsteren, A. DiNola, J. R. Haak, *J. Chem. Phys.* **1984**, *81*, 3684-3690.

[12] R. J. Gould, K. M. Murphy, S. H. Snyder, *Proc. Natl. Acad. Sci. U.S.A.* **1982**, *79*, 3656-3660.

[13] H. Schoemaker, S. Z. Langer, *Eur. J. Pharmacol.* **1985**, *111*, 273-277.

[14] I. J. Reynolds, A. M. Snowman, S. H. Snyder, *J. Pharmacol. Exp. Ther.* **1986**, *237*, 731-738.

[15] J. A. Wagner, A. M. Snowman, A. Biswas, B. M. Olivera, S. H. Snyder, *J. Neurosci.* **1988**, *8*, 3354-3359.

[16] C. Menard, S. Pupier, D. Mornet, M. Kitzmann, J. Nargeot, P. Lory, *J. Biol. Chem.* **1999**, *274*, 29063-29070.

[17] T. Pietrangelo, L. Giampietro, B. De Filippis, R. La Rovere, S. Fulle, R. Amoroso, *Eur. J. Med. Chem.* **2010**, *45*, 4928-4933.

[18] Y. Okamiya, T. Kishimoto, K. Aoki, H. Tanabe, T. Takeshita, T. Naruchi, *Eur. J. Pharmacol.* **1991**, *205*, 49-54.

[19] Z. Li, M. Nakashige, W. J. Chain, *J. Am. Chem. Soc.* **2011**, *133*, 6553-6556.

[20] a) R. M. Schelkun, P. Yuen, T. C. Malone, D. M. Rock, S. Stoehr, B. Szoke, K. Tarczy-Hornoch, *Bioorg. Med. Chem. Lett.* **1999**, *9*, 2447-2452; b) S. Menzler, J. A. Bikker, N. Suman-Chauhan, D. C. Horwell, *Bioorg. Med. Chem. Lett.* **2000**, *10*, 345-347.
